# Nonantibiotic Alternatives for Mastitis in Small Ruminants: A Scoping Review of Evidence, Gaps, and Translational Challenges

**DOI:** 10.1155/vmi/6174170

**Published:** 2026-09-21

**Authors:** Ismail Ayoade Odetokun, Damilare O. Famakinde, Rodhiat Oyinlola Ade-Yusuf, Afisu Basiru, Mutiat Adenike Adetona, Jennifer Cole, Mabrouk Elsabagh, Elena Garcia-Fruitós, Anna Arís, Mahmoud Eltholth

**Affiliations:** ^1^ Department of Veterinary Public Health and Preventive Medicine, University of Ilorin, Ilorin 240003, Nigeria, unilorin.edu.ng; ^2^ Royal Holloway University of London, Egham Hill, Egham Surrey, TW20 0EX, UK; ^3^ Department of Veterinary Physiology and Biochemistry, University of Ilorin, Ilorin 240003, Nigeria, unilorin.edu.ng; ^4^ College of Agriculture, University of Al Dhaid, Al-Dhaid, Sharjah, UAE, agriculturecollegeindore.com; ^5^ Ruminant Production Programme, Institute of Agrifood Research and Technology, Caldes de Montbui 08173, Spain; ^6^ Hygiene and Preventive Medicine Department, Faculty of Veterinary Medicine, Kafrelsheikh University, Kafr El-Sheikh, Egypt, kfs.edu.eg

## Abstract

Small‐ruminant mastitis is a marginalized yet significant challenge with multifaceted impacts on dairy production, animal welfare, economic sustainability, and public health. Reliance on conventional antibiotics to tackle the disease raises concerns about antimicrobial resistance (AMR) and food safety, especially amid the globally rising demand for small‐ruminant dairy products. Nevertheless, existing research on nonantibiotic alternatives for mastitis in sheep and goats remains poorly synthesized. To consolidate the emerging alternatives and evidence gaps, this review systematically identified relevant peer‐reviewed publications from January 2015 to October 2025. Fifteen out of the 963 records yielded were eligible. Eligible studies originated from nine countries across three continents, with notable absences in regions where small‐ruminant production coexists with high mastitis burden. Mastitis was studied under either naturally occurring or experimentally induced conditions. *Staphylococcus aureus* dominated experimental challenge models, whereas non‐aureus staphylococci were more epidemiologically relevant but less represented. A total of 20 nonantibiotic alternatives were identified across the studies, comprising phytochemicals (70%), probiotics (15%), and immunostimulants (5%). Despite their demonstrated potential to reduce antibiotic use in mastitis management, their translational relevance is constrained by factors including predominance of in vitro studies, limited pathogen identification in vivo, infrequent safety assessments, and limited benchmarking against standard‐of‐care antibiotics. Overall, the current evidence base is fragmentary but nevertheless promising. We believe that integrating strategies including standardizing research design, extending research and funding into underrepresented regions, prioritizing high‐powered pathogen‐aligned in vivo studies, and incorporating antibiotic comparators and safety evaluations will advance research toward developing sustainable, context‐specific nonantibiotic alternatives to promote antimicrobial stewardship in the management of mastitis in small ruminants.

## 1. Introduction

Small ruminants, primarily domesticated sheep (*Ovis aries*) and goats (*Capra hircus*), are economically significant herbivores raised majorly for meat, milk, fiber, and skin products. They are defined by their ability to convert low‐quality, fibrous forage into high‐quality protein, making them relatively easy to manage [1]. Historically, the domestication of sheep and goats is fundamentally intertwined with human trajectory, originating roughly 10,500 years ago [2, 3]. Sheep and goats were the first ruminants to be domesticated by humans; their domestication spread globally after first appearing in the archaeological record in Southwest Asia [2, 3]. Based on recent data, around 85% of the world’s sheep population and 96% of the world’s goat population today are found in Asia (43.6% of sheep and 55.4% of goats), Africa (30% of sheep and 38.7% of goats), and Europe (11.2% of sheep and 1.7% of goats) [4].

Sheep and goats play an important role in economic stability and sustainable livelihoods in many developing countries, with more than 330 million farmers in Africa and Asia relying on these animals for their livelihood [5]. The low capital investment required, high versatility of the animals, their natural ability to withstand extreme conditions and adapt to challenging environments, and ability to yield quick returns make them preferred over cattle, especially by small‐scale and marginal farmers in arid and resource‐poor regions [6]. At the same time, they contribute significantly to agricultural Gross Domestic Product (GDP) in many countries around the world [4, 7].

Milk is a nutrient‐dense food product that is highly beneficial for human consumption [8]. After cattle and buffaloes, small ruminants are the major contributors of dairy products, contributing to 3.5%–4.4% of the world’s milk [4, 9]. Milk produced from sheep and goats offers higher nutritional and health benefits than cow’s milk, and they are more suitable for consumption by some individuals with special dietary requirements [10, 11]. It was estimated that up to 31.4 million metric tons of milk were produced by dairy sheep and goats in 2020 [12], with about 79% of the annual global production taking place in Africa and Asia [4]. These values are, however, probably underestimated due to many unreported local productions for home consumption [13]. In many local contexts, such as in Bangladesh, for example, small ruminants may contribute up to 62% of the total milk production, reaching far more than the yield from local cattle and buffaloes [14].

While demand for small‐ruminant milk is rising, especially in Africa and Asia, where rapid population growth, increasing nutritional need, rising incomes, and urbanization are the major driving factors [15], udder diseases that impact milk yield and quality in these animals are becoming a major challenge. Among these diseases, mastitis is the most impactful, being the leading cause of economic loss and death associated with udder diseases in ruminants [16].

Mastitis in ruminants is an inflammatory disease of the mammary gland and udder tissues caused mainly by bacteria. These microbial pathogens may be spread from animal to animal (e.g., *Staphylococcus aureus* and *Streptococcus agalactiae*) or contracted from the environment (e.g., *Escherichia coli* and *Streptococcus uberis*) [17]. The intramammary inflammation can be clinical or subclinical; clinical mastitis usually accounts for < 5% of the cases, while subclinical cases are more common, persist for longer periods, and are not easily detected [17]. The covert nature of subclinical mastitis makes it a serious issue. Animals with subclinical mastitis act as reservoirs of infections, spreading bacterial pathogens to other animals. The hidden persistent inflammation of the mammary gland also reduces milk quality without visible milk changes [18]. The low milk quality is detectable during milk testing but renders the product unsuitable for sale [17, 18]. The prevalence rate of subclinical mastitis in small ruminants varies significantly between settings but may reach 32% in sheep [19] and more than 50% in goats [20, 21]. Factors that predispose animals to infection include parity, lactation stage, litter size, breed or genetics, poor housing and hygiene, poor milking practices, lack of teat disinfection, and tick infestation [17, 21, 22]. In addition to reduced milk yield, which can drop by 25%–100% [17], mastitis generally affects the animal’s welfare, results in high veterinary costs, increased use of antibiotics, and animal culling, leading to substantial economic losses [23]. Although the global magnitude of the economic loss due to mastitis in small‐ruminant production is unknown due to insufficient research attention, infrequent oversight, and limited milk marketing practices in the sector, an estimated USD 35 billion is lost globally to mastitis each year in dairy cows [17].

Antibiotics play a significant role in the treatment of mastitis. While the specific history of early clinical trials on the use of antibiotics to treat mastitis in goats (caprine mastitis) and sheep (ovine mastitis) is scarce, the practice is generally believed to have followed the adoption of antibiotic therapies for bovine mastitis in the early 1940s [24]. However, only a few drugs are licensed or labeled for use in small ruminants [25]. In the search for quick remedies, small‐ruminant farmers commonly purchase from lay outlets and use antibiotics labeled for other species, often without veterinary guidance [25]. Additionally, many smallholders raise small ruminants as a side interest, especially when the herds enlarge beyond immediate family use. As a result, they are usually not adequately informed about antibiotic dosage, frequency and route of administration, therapy withdrawal period before human use of animal products, or the potential consequences of drug residues in milk [26]. The potentially high level of indiscriminate use or misuse of many antibiotics among small‐ruminant farmers may have contributed significantly to the development of drug‐resistant bacteria in these animals. The pathogens carry high antimicrobial resistance (AMR) profiles, showing high‐level resistance to various classes of antibiotics. In a study from Kenya, over 93% of *S. aureus*, coagulase‐negative staphylococci (CoNS), and *E. coli* bacteria isolated from goat milk samples were found to have developed resistance to oxacillin [27]. Multidrug resistance was also observed in *E. coli*, *Enterobacter* spp., and *Klebsiella oxytoca* isolates [27]. Similarly, in a study from Romania, 97.3% of the isolates from sheep milk were resistant to at least two antibiotics, and 89.2% showed multidrug resistance to antibiotics including lincomycin, macrolides, β‐lactams, tetracyclines, and aminoglycosides [28]. In another study, *S. aureus* isolated from subclinical ovine mastitis showed > 73% resistance to polymyxin B and novobiocin, while isolated *Streptococcus* spp. and *Enterococcus* spp. showed absolute (100%) resistance to streptomycin, novobiocin, and tetracycline [29]. Treatment failure is also strongly linked to poor pharmacokinetics/pharmacodynamics (PD/PK) in the mammary gland environment and biofilm formation by major pathogens, especially *S. aureus* and CoNS [30].

Antibiotic use (ABU) and AMR in small‐ruminant mastitis, apart from having significant impacts on food security and economic sustainability, can directly impact human health. The presence of antibiotic residues in dairy milk following mastitis treatment [31–33] and the isolation of zoonotic antibiotic‐resistant bacteria in dairy milk [27, 28] are of particular concern. This clearly indicates that, through consumption of dairy milk, humans can ingest drug residues that may cause dysbiosis of gut microbiota and facilitate the appearance of resistant bacteria. Through the same food chain, humans can directly acquire drug‐resistant bacteria. Considering the increasing demand for small‐ruminant milk, coupled with the annual 4.71 million human deaths associated with bacterial AMR and the projected 200% increase in such deaths by 2050 [34], the potential contributory impact of ABU in small ruminants on public health is of increasing concern. To mitigate the adverse consequences, there is a pressing need to limit ABU and AMR through the development of alternative nonantibiotic strategies to control mastitis in these animals. Many potential alternatives, including phytochemicals, probiotics, nanotherapy, bacteriophages, antimicrobial peptides, homeopathy, and photodynamic therapy are being explored for mastitis control in cattle research [35, 36], but similar studies for small ruminants are less common. This scoping review aims to map and synthesize existing evidence on alternatives to antibiotics for mastitis treatment in small ruminants, specifically sheep and goat. To explore our aim, the following research questions were formulated:

1. What are the major bacterial pathogens associated with mastitis in small ruminants?
2. What types of nonantibiotic alternatives have been investigated for mastitis in small ruminants over the past decade?
3. How are these alternatives evaluated?
4. What evidence exists regarding their effectiveness and safety?
5. What gaps and translational challenges are evident in literature?

## 2. Methods

### 2.1. Study Design

We conducted a scoping review to address our main research questions. This scoping review followed the methodological framework outlined by Arksey and O’Malley [37], which includes defining a research question, conducting a comprehensive search for relevant studies, selecting appropriate studies, charting data, synthesizing information, and ultimately reporting the findings. We also adopted Preferred Reporting Items for Systematic Reviews and Meta‐Analyses guidelines extended for use with scoping reviews (PRISMA‐ScR) [38]. All protocols were developed and critically revised before commencing the scoping review.

### 2.2. Data Sources and Search Strategy

A comprehensive search for published scientific articles on alternative, nonantibiotic treatments for mastitis in sheep and goats was performed using PubMed, Scopus, Embase, Google Scholar, and Web of Science. Our search spanned studies published between January 2015 and October 2025. We integrated relevant search keywords, free texts, and Boolean operators, with modifications to suit specific databases. The search query was generally structured as follows: TITLE‐ABS‐KEY (natural^∗^ OR additive^∗^ OR probiotic^∗^ OR prebiotic OR bacteriophage OR plant^∗^ OR extract^∗^ OR herbs OR immunomodulator OR nano OR aminopeptide) AND TITLE‐ABS‐KEY (mastitis^∗^ OR udder OR “mammary gland” OR “mammary infection” OR “ovine mastitis” OR “caprine mastitis”) AND TITLE‐ABS‐KEY (sheep OR goat) AND PUBYEAR > 2014 AND PUBYEAR < 2026. A complete list of the search terms used for each database is available in Supporting Information 1. All retrieved articles were exported into Rayyan management software [39] to screen and remove or resolve duplicate publications.

### 2.3. Eligibility and Study Selection Process

The criteria for study eligibility, as clearly outlined in Table 1, were based on a Population, Concept, Context (PCC) framework, adapted from the recommendation by the Joanna Briggs Institute for scoping reviews [40]. Briefly, the population of interest for this study are small ruminants, specifically sheep and goats. Our concept is based on alternatives to antibiotics, in the context of mastitis. Only articles written in English and accessible in full text were included. Studies published before January 2015 or after October 2025 were excluded from this review. Selection of eligible studies was done in two stages. At the primary stage, two reviewers (R.O.A and M.A.A) independently evaluated the studies based on title and abstract, guided by the predetermined eligibility criteria to ensure consistency and reliability. Studies with titles and or abstracts that did not align with the research focus were discarded. Following the initial screening, both reviewers compared their findings. In the process, a third reviewer (A.B) served as an arbiter to resolve discrepancies. The second selection stage, based on full‐text screening, was performed by the same two independent reviewers. Discrepancies in the assessment were resolved through discussion between the two reviewers. Where persistent disagreements occurred, the third reviewer was invited to facilitate consensus.

**TABLE 1:** Population, Concept, Context (PCC) framework for study eligibility on nonantibiotic alternatives in the treatment of mastitis in small ruminants.

	Inclusion criteria	Exclusion criteria
Population	• Articles strictly involving sheep and goats worldwide.	• Original articles on animals excluding sheep and goats. • Review articles and case reports.

Concept	• In vivo or in vitro studies focused on exploring alternatives to antibiotics.	• Studies focused on conventional or other antimicrobial drugs.

Context	• Studies focused on mastitis, udder diseases, udder infection, or udder inflammation. • In vitro studies that experimented with bacterial isolates from mastitis cases. • In vitro studies that experimented with nonclinically isolated bacteria or bacterial products on mammary tissue or cells	• Studies addressing diseases unrelated to udder health. • In vitro trials that used mastitis‐causative bacteria that were not isolated clinically. • In vitro trials that used nonclinically isolated bacteria or bacterial products on nonmammary tissues or cells.

### 2.4. Quality Appraisal of Included Studies

To assess the quality of the articles included in this review, we adopted the Critical Appraisal Skills Program (CASP) checklist tool [41]. The checklist comprised 10 questions which we modified slightly to accommodate the type of research studies included. For in vivo studies, the following checklist questions were asked: (i) Did the study address a clearly formulated research question? (ii) Were the experimental animals’ source and pedigree well described? (iii) Was the assignment of animals to intervention groups randomized? (iv) Were the groups similar at the start of the randomized controlled trial (weight, age, and health)? (v) Was the alternative to antibiotics used clearly stated and authenticated? (vi) Was the treatment method used clearly stated and did it follow standard methods? (vii) Did the ethics review board approve the research and was the board mentioned? (viii) Was there a clearly defined study protocol (were the parameters measured standard and clearly defined? (ix) Were the effects of intervention reported comprehensively? (x) Were all the animals used in the study accounted for at its conclusion? For in vitro studies, the checklist entailed the following questions: (i) Was the research objective well stated? (ii) Was the study on alternatives to antibiotics in management of mastitis‐causing organisms? (iii) Were there replicates? (iv) Was the alternative to antibiotics well stated and authenticated? (v) Ethical approval obtained? (vi) Were the sources of the isolates used clearly described? (vii) Were the pathogen identification methods well described and according to standard procedure? (viii) Were the positive and negative controls stated? (ix) Were the outcomes clearly stated and measured according to the standard procedure? (x) Were the results clearly presented? By allocating 1 for “Yes” and 0 for “No,” each article was scored per question, with the highest possible total score of 10. The percentage score was calculated for each article. Articles that had a total score of ≥ 50% were recruited for data analysis and result writing. This threshold falls within the 40%–70% range that is usually considered moderate [38]. Quality assessment was performed independently by the same two reviewers (R.O.A and M.A.A). Conflicts were resolved by mutual discussions between both reviewers and by the intervention of the third reviewer (A.B).

### 2.5. Data Charting and Reporting of Results

The charted data from each record included but were not limited to study ID, author, year of publication, geographical location of study, study design, animal, breed, bacteria inoculated or isolated, method of bacterial inoculation, nonantibiotic alternatives used, category of the nonantibiotic alternatives, route of administration of the alternatives, main variables tested, main outcomes, efficacy, percentage risk‐of‐bias scores, research funding, and references. A data extraction table (Supporting Information 2) was collaboratively developed by our team members. Each independent reviewer was assigned a part of the data extraction. All extracted data were further refined and calibrated with a high level of agreement among the reviewers. In the case of missing data or unclear pieces of information, it was considered that the authors did not report such variables. Studies where subclinical or clinical mastitis status was not confirmed in the ruminants before commencing intervention treatment were considered prophylactic. Although in vitro studies are almost universally used to determine pathogen susceptibility and guide therapeutic decisions, based on the experimental timing (not the experimental intent), we modeled in vitro studies as prophylactic (intervention treatment first + bacterial inoculum) or therapeutic (bacterial inoculum first + intervention treatment). All results were coherently presented in both descriptive and narrative summaries. For visual representation of the search methods and result findings, Microsoft 365 Apps (Microsoft Corporation, Redmond, WA, USA), sankeyMATIC (https://sankeymatic.com), BioRender (https://www.biorender.com), and R software (R v4.3.2) [42] were used. All codes and scripts used in data visualization can be accessed via GitHub at https://github.com/DamiF25/BAC4RumA_SmaRT.git.

## 3. Results

### 3.1. Publication Screening Outcomes

**FIGURE 1:**
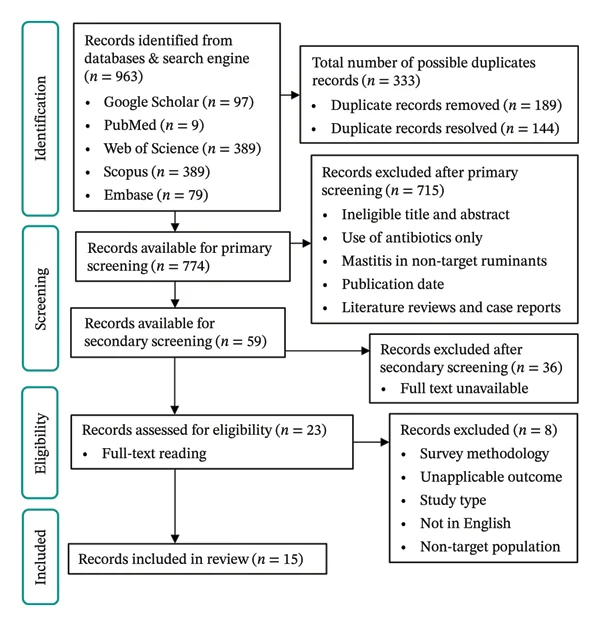
PRISMA workflow diagram for a scoping review on alternatives to antibiotics use for mastitis management in small ruminants.

A total of 963 articles were retrieved from five sources including Google Scholar (97), PubMed (9), Web of Science (389), Scopus (389), and Embase (79). Duplicate publications were detected, resolved, and deleted, leaving 774 articles to be screened. After primary screening, 715 articles were further excluded. After the secondary full‐text screening of the remaining 59 articles, only 15 articles met the final eligibility criteria (Figure 1). All the 15 articles passed study quality appraisal, with scores ranging between 50% and 100% for in vivo studies and between 70% and 90% for in vitro studies (Supporting Information 2).

### 3.2. General Trends and Study Characteristics

**FIGURE 2:**
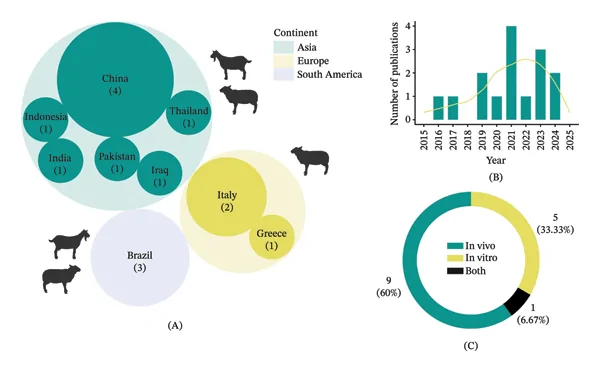
General overview of the included studies showing (A) the demographic distribution, (B) the number of publications per year within the period covered, and (C) the proportion of studies that were performed in vivo and in vitro.

All the 15 eligible studies were conducted in nine countries across Asia (including the Middle East), Europe, and South America (Figure 2A). The larger proportion (60%, *n* = 9) of the studies were conducted in Asia, and studies from South America emanated only from Brazil. By country, China had the highest number of publication records (26.7%, *n* = 4), followed by Brazil (20%, *n* = 3) and Italy (13.3%, *n* = 3). Each of the other countries (Indonesia, India, Thailand, Pakistan, Iraq, and Greece) had a single publication record (6.7%, *n* = 1). Although the total number of eligible studies was limited, annual progression showed a clear growth in research interest on nonantibiotic alternatives for mastitis in small ruminants over time, especially between the years 2019 and 2024, with no eligible studies found in 2015, 2018, and during the period covered in 2025. The year 2021 alone yielded 26.7% of all included studies (Figure 2B).

Out of the 15 records included in this review, 9 records (60%) were performed in vivo only, 5 records (33.3%) were performed in vitro only [43–47], and 1 record (6.7%) combined both in vitro and in vivo studies [48] (Figure 2C). The in vivo studies comprised six (40%, *n* = 6) caprine studies [49–54] and three (20%, *n* = 3) ovine studies [55–57] (Supporting Information 2). Whether in vitro or in vivo, studies from Asia and South America involved sheep and goats, whereas studies from Europe were exclusively conducted on sheep (Figure 2A) (Supporting Information 2). Most of the studies did not clearly state animal age. From the available data, goats of 1.25–4 years of age were recruited. Various breeds of goats, including Guanzhong (China), Saanen (China and Brazil), Bengal (India), Etawah crossbreed (Indonesia), and Beetal (Pakistan), and various breeds of sheep including Chios (Greece), Sarda, Valle del Belice, and Comisana (Italy) were used (Supporting Information 2).

### 3.3. Overview of the Bacterial Pathogens Associated With Mastitis in Small Ruminants

**FIGURE 3:**
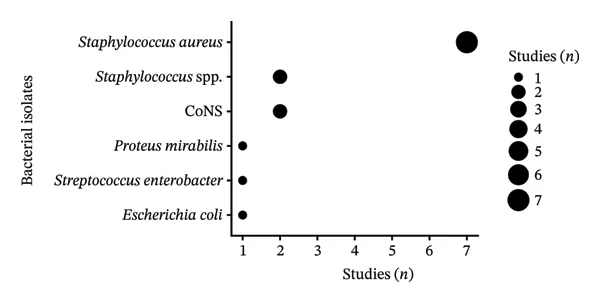
Bacterial isolates and number of studies from where they were reported. CoNS: coagulase‐negative staphylococci.

It is important to emphasize here that some studies recruited clinically healthy sheep and goats, potentially with subclinical mastitis, but did not isolate milk bacteria or report their taxonomic identity when isolated. Hence, we could not identify the potential bacterial pathogens in such studies. Such studies comprised 50% of the in vivo studies, four out of the seven caprine studies (∼57%) [49, 51–53], and one of the three ovine studies (33.3%) [55]. From a total of nine studies (in vivo and in vitro) where the identity of bacterial pathogens was known, *S. aureus* was reported or used in 77.8% (7/9) of the studies (Figure 3). CoNS were identified with mastitis‐associated cases in two studies [43, 57]. One of the studies further identified the CoNS in lactating ewes as *S. capitis*, *S. caprae*, *S. caseolyticus*, *S. chromogenes*, *S. epidermidis*, *S. haemolyticus*, *S. lentus*, *S. saccharolyticus*, *S. sciuri*, *S. simulans*, *S. warneri*, and *S. xylosus*. The coagulase‐variable *S. hyicus* was also isolated [57]. Other staphylococci were reported but not at the species level. Other than *Staphylococcus*, pathogenic bacteria including *Escherichia coli*, *Proteus mirabilis*, and *Streptococcus enterobacter* were also implicated in caprine and ovine mastitis (Figure 3).

### 3.4. The Nonantibiotic Alternatives Tested

**FIGURE 4:**
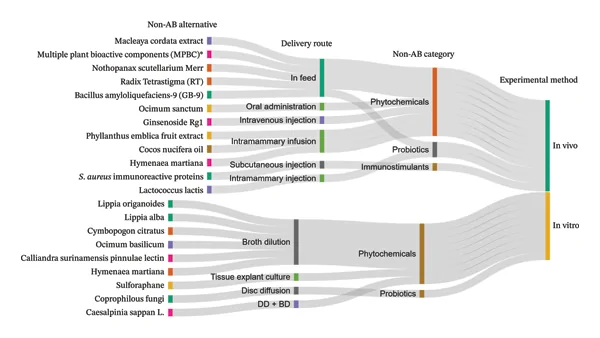
The nonantibiotic (non‐AB) alternatives used in vivo and in vitro, including their category and route of delivery. DD + BD: combined disc diffusion and broth dilution; ^∗^MPBC is a mixture of plant bioactive components: thyme (*Thymus vulgaris*), anise (*Pimpinella anisum*), and olive (*Olea europea*).

We identified a total of 20 nonantibiotic alternatives, which we grouped under three different categories, namely, phytochemicals, probiotics, and immunostimulants (Figure 4). Of these alternatives, 11 were tested in vivo only, 8 were tested in vitro only, and 1 was tested both in vitro and in vivo (Figure 4). Generally, the use of phytochemical‐based alternatives was more common, being used in 83.3% (10/12) and 88.9% (8/9) of the in vivo and in vitro studies, respectively (Figure 4). Probiotics were used in vivo (*Bacillus amyloliquefaciens*‐9 and *Lactcoccus lactis*) and in vitro (coprophilous fungi), while immunostimulants (*S. aureus* immunoreactive proteins) were used only in vivo. In terms of their delivery route, a larger number (5/12) of the alternatives administered in vivo were added to feed, followed by the intramammary infusion method (3/13). Other routes of in vivo delivery included oral administration, intravenous injection, subcutaneous injection, and intramammary injection. Altogether, six different methods of in vivo delivery were found (Figure 4). In vitro, alternatives were mostly tested through broth dilution (6/9) and less commonly through disc diffusion, tissue explant culture, and the combination of disc diffusion with broth dilution (Figure 4).

### 3.5. Infection Sources and Intervention Approaches

**FIGURE 5:**
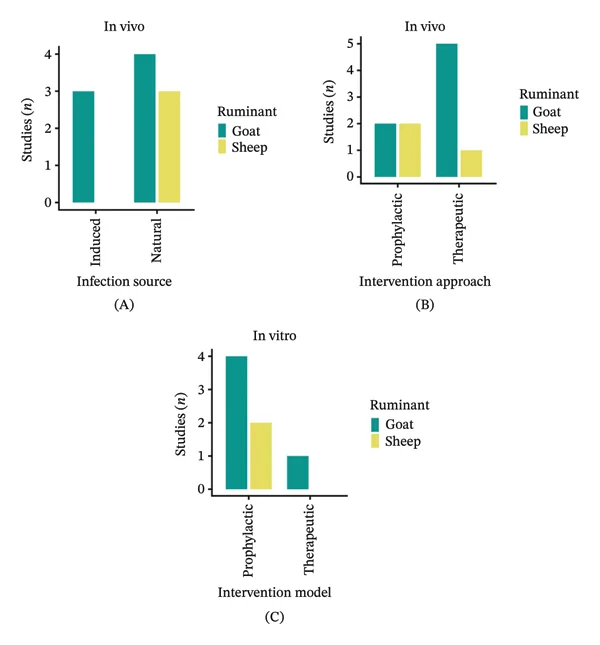
Infection sources and approaches to the delivery of nonantibiotic interventions in caprine (goat) and ovine (sheep) studies on mastitis. (A) Number of in vivo studies that experimentally induced mastitis or worked with natural mastitis infection in goats and sheep. (B) Number of in vivo studies that administered the nonantibiotic alternatives prophylactically or therapeutically in sheep and goats. (C) Number of in vitro studies that modeled prophylactic or therapeutic approaches in caprine and ovine studies.

Bacterial mastitis infections in the experimental sheep and goats were identified as induced (when known bacterial pathogens or their products were deliberately introduced into the animals to establish mastitis) or natural (when the infection was not experimentally induced). Natural infection sources included studies that recruited clinically healthy sheep and goats with potential subclinical mastitis, even when the milk bacterial pathogens were not isolated or reported. In all caprine studies where pathogens were identified (∼43%, 3/7), mastitis was experimentally induced in the goats through intracisternal inoculation or intramammary infusion [48, 50, 54]. In contrast, all ovine studies recruited naturally mastitic sheep (Figure 5A). All in vitro studies were basically experimental, inoculating bacterial isolates from prior caprine or ovine mastitis cases.

Generally, the nonantibiotic alternatives were administered prophylactically (58.8%) or therapeutically (41.2%). In goats, therapeutic approaches were used more frequently (∼71%, 5/7), while in sheep, prophylactic approaches were slightly more common (66.7%, 2/3) (Figure 5B). In vitro, prophylactic models were common in caprine studies (80.0%, 4/5) (Figure 5C). In a prophylactic model, for example, lactating goat mammary epithelial cell culture was pretreated with sulforaphane, a phytochemical isothiocyanate compound, before introducing mastitis‐inducing bacterial lipopolysaccharide [47]. Only one in vitro study was found in ovine studies, modeling the therapeutic approach (Figure 5C).

### 3.6. Outcomes on the Efficacy of the Nonantibiotic Alternatives

The experimental variables examined to determine the efficacy of the alternatives in the small ruminants generally included, but not limited to, postintervention bacterial clearance, milk somatic cell count (SCC), milk yield and quality, pH, California mastitis test (CMT) scores, lactoperoxidase (LPO) activity and alpha‐lactalbumin level, malondialdehyde (MDA) and N‐acetyl‐β‐d‐glucosaminidase (NAGase) levels, antioxidant activity (such as catalase and glutathione peroxidase levels), serum inflammatory indicators (such as IFN‐γ and IL‐6), udder size, mammary tissue fibrosis, and supramammary lymph node swelling. In the in vitro context, bactericidal/bacteriostatic effects, antibiofilm activity, antioxidant activity, and regulatory gene expression effects of the alternatives were assessed to determine efficacy. Some studies also examined the potential adverse effects that may be associated with these alternative interventions by assessing additional parameters, including blood urea nitrogen (BUN or PUN), serum aspartate aminotransferase (AST)/glutamic oxalacetic transaminase (GOT/SGOT), creatinine (CRT) levels, feed intake, gut microbiota dysbiosis, allergies, behavioral changes, cytotoxicity (also for ex vivo experiments), and animal mortality. Table 2 summarizes the research outcomes on each alternative tested.

**TABLE 2:** Reported efficacy of the various nonantibiotic alternatives against mastitis in sheep and goats.

		**Nonantibiotic alternative**	**Dose/concentration**	**Duration/dosage**	**Results**	**Reference**
**In vivo**	Bacteria or bacterial product inoculated**^1^**/isolated**^2^**					
Goat	NA. Dairy goats were recruited but no bacteria were inoculated/isolated	*Macleaya cordata* extract	400 mg/kg of diet	52 days	Lower milk somatic cell count (SCC) (*p* < 0.05); increased milk yield (*p* < 0.05); increased alpha‐lactalbumin level (*p* < 0.01); lower levels of BUN and AST	[52]
	*Staphylococcus aureus* ^1^	*Ocimum sanctum* leaf juice	5 mL/kg of body weight	14 days	Enhanced immune defense; reduced SCC, negative CMT; elimination of *S. aureus* colonies; improved milk enzyme activity (all *p* < 0.05) No changes in AST, PUN, and CRT levels	[50]
	NA. Lactating goats were recruited. Milk bacteria were counted but the isolated species were not reported	*Nothopanax scutellaium* Merr (NSM) leaves	20 g per day	Not clearly stated	Lower milk SCC; lower milk bacteria; increased milk yield (all *p* < 0.05); increased feed intake	[49]
	*Escherichia coli* cell wall lipopolysaccharide^1^	Ginsenoside Rg1	2.5 mg/kg of body weight	Two injections at 2 and 4 h postchallenge	Significant reduction of inflammatory responses, milk SCC, and NAGase activity, while preserving milk yield and restoring systemic immune parameters (all *p* < 0.05)	[54]
	NA. Mastitic lactating goats were recruited. Bacteriological investigation was carried out but milk bacterial isolates were not reported	*Phyllanthus emblica* fruit extract	1500 mg in 10 mL normal saline solution per day	5 days	Reduced milk pH, SCC, and ALP; reduced CMT; increased milk yield; similar cure rates compared with standard antibiotics	[53]
	NA. Same as above	*Cocos nucifera* oil	10 mL per day	5 days	Same as above	[53]
	NA. Mastitic dairy goats were recruited but no milk bacteria were isolated	*Bacillus amyloliquefaciens*‐9 (GB‐9)	0.3% (w/w) of total mixed ration	6 weeks	Reduced SCC; reduced IgA, IgM, IL‐2, IL‐4, and IL‐6; reduced MDA (all *p* < 0.05) Fecal microbiota: Relative abundance of *Akkermansia* and *Phascolarctobacterium* but reduction in *Bacteroides* and *Prevotellaceae*	[51]
	NA. Same as above	*Radix tetrastigma*	Same as above	6 weeks	Reduced SCC; reduced IgA, IgM, IL‐2, IL‐4, and IL‐6; reduced MDA (all *p* < 0.05); fecal microbiota: relative abundance of *Succinivibrio* and *Butyrivibrio* but reduction in *Prevotellaceae*	[51]
	*S. aureus* ^1^	*Hymenaea martiana* (jatobá) leaf crude ethanol extract (CEE)	5% CEE	6 days	Similar cure rates compared with standard antibiotics. 5% CEE regressed disease in 4/5 treated mammary halves, while gentamicin regressed 5/5 treated mammary halves	[48]

Sheep	NA. Ewes were grouped based on their milk yield and body weight. No bacteria were inoculated/isolated	MPBC	0.025% and 0.05% of diet	11 weeks	Lower milk SCC; increased milk yield; improved milk oxidative stability (all *p* < 0.05)	[55]
	*S. aureus* ^1^	*S. aureus* immunoreactive proteins	Not stated	45 days (one injection per 15 days)	Strong immunogenicity and humoral IgG response	[56]
	CoNS*, S. aureus* ^2^	*Lactococcus lactis*	3 mL of ∼1 × 10^8^ CFU/mL	Once daily for 3 days (study 1) and 7 days (Study 2)	Significantly increased SCC (*p* < 0.05); strong mammary immune activation but failure to sustain bacteriological cure; abnormal milk (flakes, watery secretion); 40% of CoNS‐infected glands developed mild to moderate clinical mastitis and 30% of *S. aureus*‐infected glands showed worsening clinical signs	[57]

**In vitro**	Bacteria or bacterial product tested**^∗^**					

Goat	*S. aureus*, *Staphylococcus* spp.	*Lippia origanoides*	25–3200 μg/mL	24–48 h	Total (100%) inhibition of the bacterial isolates at a mean; minimum inhibitory concentration (MIC) of 560 μg/mL	[44]
	*S. aureus*, *Staphylococcus* spp.	*Lippia alba*	Same as above	Same as above	Total (100%) inhibition of the bacterial isolates at a mean MIC of 1173 μg/mL	[44]
	*S. aureus*, *Staphylococcus* spp.	*Cymbopogon citratus*	Same as above	Same as above	Total (100%) inhibition of the bacterial isolates at a mean MIC of 1280 μg/mL	[44]
	*S. aureus*, *Staphylococcus* spp.	*Ocimum basilicum*	Same as above	Same as above	Inhibited 0% of the bacterial isolates	[44]
	*S. aureus*, *Staphylococcus* sp. Ssp.	*Calliandra surinamensis* leaf pinnulae lectin	0.03–15.0 μg/mL	GIAs: 24 h GCs: 6 h BAs: 24 h	Strong bacteriostatic effects on *Staphylococcus* Ssp5D and Ssp01 isolates; antibiofilm activity *S. aureus* and one *Staphylococcus* sp. isolate at sub‐inhibitory concentrations	[46]
	Bacterial lipopolysaccharide	Sulforaphane	1.25, 2.5, and 5 μmol/L	12–24 h	Downregulated expression of inflammatory factors (*p* < 0.05); increased antioxidant effect (*p* < 0.05); low cytotoxicity	[47]
	*S. aureus*	*H. martiana* (jatobá) leaf CEE	97.6–12,500 μg/mL	24 h	Strong antibacterial and antibiofilm activity, with lowest MIC of 195.3–781 μg/mL	[48]
	CoNS	*Caesalpinia sappan*	0.195–100 mg/mL	24–42 h	Ethanol extracts of *C. sappan* showed strong antimicrobial effects at MIC of 1.56 mg/mL	[43]

Sheep	*S. aureus*, *Staphylococcus* spp.	*Lippia origanoides*	25–3200 μg/mL	24–48 h	Inhibited 100% of the bacterial isolates at a mean MIC of 560 μg/mL	[44]
	*S. aureus*, *Staphylococcus* spp.	*Lippia alba*	Same as above	Same as above	Inhibited 100% of the bacterial isolates at a mean MIC of 1173 μg/mL	[44]
	*S. aureus*, *Staphylococcus* spp.	*Cymbopogon citratus*	Same as above	Same as above	Inhibited 100% of the bacterial isolates at a mean MIC of 1280 μg/mL	[44]
	*S. aureus*, *Staphylococcus* spp.	*Ocimum basilicum*	Same as above	Same as above	Inhibited 0% of the bacterial isolates	[44]
	*S. aureus, E. coli, Streptococcus enterobacter, Proteus mirabilis*	Crude extract of *Coprophilous* fungi	Not clearly stated	24 h	Significant (*p* < 0.05) antibacterial activity against mastitis‐associated pathogens, depending on fungal species, extraction solvent, and tested bacteria	[45]

*Note:* AST: serum aspartate aminotransferase; Bas: biofilm assays; BUN/PUN: blood/plasma urea nitrogen; CRT: creatinine; MPBC: mixture of plant bioactive components (*Thymus vulgaris*, *Pimpinella anisum*, and *Olea europea*).

Abbreviations: CFU, colony‐forming unit; CMT, California mastitis test; CNS, coagulase‐negative *Staphylococci*; GCs, growth curves; GIAs, growth inhibition assays.

^1^Inoculated into healthy ruminants to induce mastitis.

^2^Isolated from naturally infected ruminants.

^∗^All bacteria tested in vitro were isolated from previous mastitis cases; the bacterial product (lipopolysaccharide) was commercially sourced but used on goat mammary epithelial cell culture.

### 3.7. Standard Antibiotic Benchmarking

**FIGURE 6:**
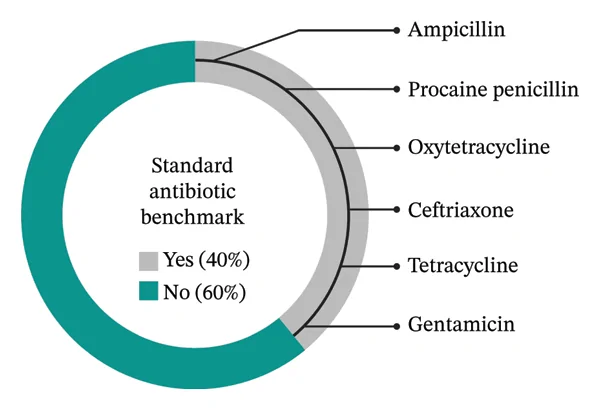
The proportion of studies that included standard antibiotic comparators.

Less than half (40%) of the total studies (both in vitro and in vivo) included a control experiment to weigh the effectiveness of the alternatives relative to conventional antibiotics. In total, six antibiotics under four different pharmacological classes were used as standards. These were penicillins (ampicillin and procaine penicillin), tetracyclines (tetracycline and oxytetracycline), cephalosporins (ceftriaxone), and aminoglycosides (gentamicin) (Figure 6).

## 4. Discussion

This study was conceived to evaluate the research landscape and emerging evidence on nonantibiotic alternatives to reduce ABU in small‐ruminant mastitis over the past decade. Although research interest in small‐ruminant mastitis may be generally high, directly relevant evidence on alternatives to its antibiotic treatment remains narrow, considering the paucity of study records and heterogeneity in study designs and outcome measures. This narrowness is not a failure of individual studies but may reflect how infrequent small ruminants are incorporated into the global conversation on antimicrobial stewardship and alternative therapies or how limited the global investment is in this domain. This gap points to a clear priority for policymakers and funding bodies. Often, national and regional AMR action plans generically refer to “livestock,” but the research and funding pipelines overwhelmingly favor the cattle sector, followed by poultry, piggery, and aquaculture sectors [58]. Although research priorities and funding are directed into livestock sectors where potential economic gains are most enormous, it should be noted that small ruminants are the silent workhorses of many rural economies and livelihoods. As such, they deserve a proportional share of research investment. International donors and development agencies could appropriate funds for small‐ruminant mastitis research in under‐represented regions, especially Africa and parts of Asia where smallholder systems dominate and antibiotic access is often unregulated.

Ultimately, we questioned several aspects of bacterial mastitis in small ruminants, including what the main pathogens are, what nonantibiotic alternatives are being evaluated against them and through what approaches, whether the alternatives can realistically reduce ABU, and what the impedances to their implementation are. The answers that emerged, as discussed in detail in this section, were, however, more complex than the questions asked.

### 4.1. A Global Blind Spot of Geographical and Species Imbalances

Despite the scanty records, spatial distribution of the included studies reveals some structural inequalities. The prominence of data from Asia and the disproportionate contribution in China reflect both continental and regional dominance in small‐ruminant populations and research investments [4]. In Europe, more research output on ovine than caprine mastitis may mirror the small‐ruminant population structure and the prevalent farming practice. The Italian sheep population is five times more than the goat population [59], and 88% of small‐ruminant (sheep and goat) farming is practiced with sheep [60]. In South America, Brazil’s exclusivity reflects its status with the largest small‐ruminant populations in the region [61] and suggests a national research ecosystem that is more responsive to mastitis in small‐ruminant health. Taken collectively, it appeared that European research prioritizes mastitis in sheep, whereas Asian and South American studies focus on both sheep and goats. These patterns may not only represent regional production systems but also highlight the need for species‐specific research, given the differences in the mammary physiology between sheep and goats. For instance, unlike in sheep, milk secretion in goats occurs through an apocrine mechanism that is accompanied by the release of cytoplasmic particles into the secreted milk, therefore, complicating SCC interpretation [62], and may also influence how alternatives perform in vivo.

While we recorded studies from Asia, Europe, and South America, we did not identify any eligible study from Africa, despite that the continent ranks second only to Asia in dairy sheep and goat populations [4], and was reported with high mastitis burden in ruminants [27, 63, 64]. This places a limitation on the generalizability of our findings because factors such as pathogen pressure, alternative therapies, production systems, diagnostic capacity, research governance, and climate in Africa may differ markedly from those in Europe, Asia, and South America. For instance, many African regions have limited research and development funding and fewer biotechnological centers. In such regions, alternative treatment may rely on locally adapted solutions, such as ethnoveterinary botanicals. As a result, the portfolio of alternatives used in Asia, Europe, and South America may not represent the innovation landscape in Africa. In the African context, how these alternatives are tested may also differ from the controlled in vivo and in vitro approaches that dominate our current dataset. Constraints in laboratory capacity and experimental facilities in many African institutions may increase the feasibility for field‐based observational trials and participatory evaluations [65, 66]. Furthermore, the confidence with which we can extrapolate the potential of these alternatives in reducing ABU may be partly weakened since the potential for the adoption of new alternatives depends on affordability, availability, regulatory approval, and farmers’ perceptions, all which may vary in the African small‐ruminant systems compared with other regions, especially for nonphytochemical candidates. In addition, the performance or practicality of the alternatives may differ due to environmental stressors that are unique to a geographical region, such as climate, seasonal feed or water scarcity, housing and shelter limitations, high environmental pathogen load, and coinfection with other pathogens or parasites. These are all factors that shape disease dynamics and may confound animal response to therapeutic interventions.

Importantly, the nonappearance of African data reflects a potential global research imbalance that risks the neglect of regions where antibiotic stewardship is most urgently needed. With such under‐representation, the gap in the global North–South research alignment widens, fostering a defective system in which discoveries and evidence are concentrated in well‐resourced regions with stronger laboratory infrastructure, more stable funding streams, established biotechnology sectors, and more technical experts [67]. Interventions that progress through such systems may lose their relevance and feasibility in low‐ and middle‐income small‐ruminant systems where they are needed most. A successful AMR reduction with new alternatives, therefore, requires geographical inclusivity, and regions with the highest disease burden should be central to innovation pathways [68]. Addressing this imbalance through strategic funding programs, North–South or South–South research partnerships, and regional capacity building in resource‐limited regions is not only a scientific necessity but also a crucial approach to ensure that future evaluations of nonantibiotic alternatives are globally representative.

Another possible implication is the risk of perpetuating knowledge invisibility for African‐led research and grassroots innovation [69, 70], rendering them unseen by global decision‐makers or policymakers. Locally developed alternatives, whether ethnoveterinary botanicals, farmer‐driven hygiene strategies, low‐cost management interventions, or crude probiotics, may remain unpublished or appear only in regional platforms that are not indexed in major research databases. This structural invisibility excludes valuable insights from global syntheses such as this present study, amplifying the perception that innovation is absent where it is only underdocumented. Strengthening regional publishing ecosystems by improving journal indexing and supporting open‐access dissemination, especially in the global south, is, therefore, an essential component of a more equitable and globally informative research landscape.

### 4.2. Pathogen Focus and Experimental Bias

One of the clearest insights from this review is the mismatch between the experimental pathogens and those that are more relevant in real‐world mastitis cases. Therefore, the predominance of *S. aureus* in the reviewed studies should be interpreted cautiously. This is because a substantial number of *S. aureus* records (both in vivo and in vitro) deliberately introduced the pathogen. It is imperative to emphasize that the frequent choice of *S. aureus* in both in vivo and in vitro experimental studies is informed by its well‐established pathogenicity and tendency to develop resistance to antibiotics, making it a significant model for assessing alternative mastitis control methods [71, 72]. However, this practice biases the evidence base away from the pathogens that most commonly drive natural subclinical mastitis in small ruminants. Epidemiological studies frequently show non‐aureus staphylococci (NAS), mainly comprising CoNS, as the main agents of subclinical mastitis in sheep and goats [19, 20, 73–75], while some other studies showed the dominance of *S. aureus* [29]. A major methodological limitation we observed, which further constrained evidence on the real pathogen diversity, was that nearly all in vivo studies that recruited sheep and goats with potential subclinical mastitis did not isolate or comprehensively identify milk bacteria compositions. As a result, improvements in SCC or clinical signs could not be confidently attributed to pathogen‐specific effects. The only caprine study that identified bacterial species in milk samples from lactating ewes also found that CoNS were disproportionately represented [57]. The mismatch in experimental versus field reality has important implications, as alternatives evaluated primarily against *S. aureus* may perform differently against NAS due to differences in their pathogenicity and virulence. Hence, this gap limits the external validity of the findings and highlights the need for future trials to tailor the study of nonantibiotic alternatives to the real pathogen landscape. Accurate and more reliable data on mastitis‐causative bacteria across various environmental and regional settings will guide national antimicrobial stewardship programs toward targeted and effective interventions, without which farmers may continue to rely on broad‐spectrum antibiotics even when alternatives could be more appropriate.

### 4.3. The Diversity, Potentials, and Limitations of the Current Alternatives

The twenty (20) nonantibiotic alternatives identified across the reviewed records represent three major categories. This diversity is narrow, especially when compared with the increasing array of alternatives that are being evaluated in bovine mastitis research, including nanotechnology‐based therapy, bacteriophage therapy, homeopathy, photodynamic therapy, acoustic pulse therapy, nonsteroidal anti‐inflammatory drug therapy, antimicrobial peptide therapy, vaccination, mesenchymal stem cell therapy, and the use of zeolites, cytokines, ozone, and CRISPR‐guided antimicrobials [30, 35, 36]. Generally, in terms of evidence maturity and clinical validation, there is a fundamental difference between small‐ and large‐ruminant mastitis research. For example, more bovine mastitis research is based on randomized controlled trials to validate some of the nonantibiotic alternatives through multidimensional data points. On the other hand, small‐ruminant records identified in this review are largely preliminary or confined to in vitro models, therefore, lacking the equivalent baseline data on herd‐level efficacy. Also, unlike in bovine mastitis where targeted interventions such as bacteriophage cocktails [76] or specific vaccine formulations [77] are common, small‐ruminant mastitis research lacks standardized protocols tailored to its unique nature. Using bovine methodologies as a benchmark could enable small‐ruminant research to transition from exploratory testing to more validated, field‐ready applications. Unarguably, most technological innovation and pharmaceutical investments are channeled toward the bovine systems and, usually, new veterinary therapies are first developed, validated, and approved for use in cattle before they are adapted to small ruminants, such as sheep and goats [78]. While such “technology transfer” model may be reasoned to help in conservative use of funds rather than conducting expensive parallel research, such system might also inadvertently delay progress in addressing mastitis in small ruminants or result in less effective control strategies. For example, despite sharing several physiological similarities, important differences still exist between cattle and small ruminants in terms of mammary physiology, immune responses, pharmacokinetic profiles, disease epidemiology, and management practices, including grazing systems and milking frequency [62, 78–81]. Going forward, targeted research efforts that integrate biological and production‐system peculiarities while exploring innovative nonantibiotic techniques for small ruminants may be helpful.

The notable dominance of plant‐derived compounds as potential alternatives to antibiotics in small‐ruminant mastitis reflects both the easy accessibility and the profound ethnoveterinary practices in small‐ruminant‐rearing regions. The alternatives evaluated (phytochemicals, probiotics, and immunostimulants) demonstrated good promise across in vitro and in vivo endpoints. However, the present literature does not offer standardized, comparative evidence that can underpin guidelines or product labels. A significant proportion of the studies were performed in vitro. Although relatively rapid, inexpensive, and indispensable in the initial screening of new therapeutic compounds, in vitro studies are unable to provide mechanistic evidence or fully replicate the complexity of a living system [82]. Therefore, results from such studies are usually restricted in their direct translation to clinical application. In vivo, the absence of standardized dosing, formulation, and varied delivery routes is a major barrier. With such inconsistencies, it is impossible to know whether an experimental failure is due to an ineffective compound, an inadequate dose, or an inappropriate intervention administration route. In the same vein, different case definitions, SCC thresholds, CMT scoring systems, follow‐up periods, and combinations of outcomes were used across the studies. In such a landscape, each study becomes an independent output, fragmenting knowledge in the field. Moreover, most alternatives were tested in small sample sizes, which may inadequately represent the dynamics under real production systems.

For a nonantibiotic alternative to be adopted widely, farmers and regulators need to be reassured that it does not compromise animal welfare, milk quality, or consumer safety. So far, the safety aspect of the alternatives evaluated for ruminant mastitis remains underdeveloped. This is because adverse events were inconsistently defined and reported. Although some studies evaluated safety parameters such as liver enzymes, creatinine, feed intake, behavior, and mortality in the experimental fish, systemic safety evaluation was rare. Data on residues that may persist in milk or meat following interventions, such as phytochemicals, are lacking. This can be generated through targeted pharmacokinetic and residue depletion studies [83, 84], which are critical for establishing withdrawal periods to ensure food safety in livestock. For regulators, the absence of systemic safety data is a red flag, and any alternative intervention that is associated with unknown risks will struggle to gain traction, even if it promises reduced ABU.

One of the most significant limitations of the current evidence base is the frequent omission of comparative trials with standard antibiotics. The absence of such comparisons hinders the evaluation of noninferiority or superiority of new alternatives [85, 86]. It becomes impossible to determine whether alternatives have better or no worse consequences on animal welfare or consumer safety, relative to standard antibiotic therapy. For veterinarians and policymakers, placing new alternatives on this clinical scale is crucial for informed decision‐making. Future research must, therefore, prioritize high‐powered, randomized, controlled trials that benchmark alternatives against standard therapies, focusing on clinically relevant endpoints including those that matter to farmers (such as cure rates and milk yield) and microbiologists (such as bacterial clearance and resistance selection). Although including antibiotic comparators may increase cost and require more ethical considerations or stricter regulatory oversight, it is yet an unavoidable requisite to move beyond the current experimental margins. One way forward could be to develop a set of core outcomes for small‐ruminant mastitis trials, agreed upon by researchers, clinicians, and farmers. This might include a minimum set of microbiological, clinical, production, and welfare outcomes, with standardized measurement methods and reporting formats. Journals and funders could also help by encouraging or requiring researchers to evaluate the basic set of parameters, while still allowing more exploratory endpoints. Over time, it will be possible to pool data, identify patterns, refine methods, and make actionable recommendations.

While the current research is more focused on affordable plant‐based alternatives, research expansion that accommodates non‐plant‐based alternatives and more advanced technologies will have to examine socioeconomic and behavioral factors, such as cost‐effectiveness and farmers’ perceptions, which may constitute barriers to future adoption, especially in many developing settings [87]. This is especially needed when alternatives are potentially more expensive, more labor‐intensive, less familiar, or require changes in management practices that are difficult to implement in smallholder systems. Failure to understand how farmers and smallholders perceive the risk, efficacy, cost, and practical feasibility of new alternatives risks developing technically sound interventions that are not embraced by the supposed end‐users.

### 4.4. Different Roles and Expectations of Prophylactic Versus Therapeutic Interventions

Another key insight from this review is the frequency of prophylactic and therapeutic approaches used. The literature shifts toward prophylactic testing, especially in ovine studies and in vitro models. Prophylactic strategies are particularly attractive in the context of small ruminants. Given that subclinical mastitis is more common and can persist undetected in these animals, any disruption in the delicate balance between the pathogen structure and the animal immune system can rapidly progress subclinical mastitis into clinically relevant cases [33, 75], causing repeated ABU. Therefore, preventing the establishment of clinical infections could meaningfully reduce ABU at the flock level. By contrast, therapeutic trials, which were more common in caprine studies, may reflect the practical challenge of treating clinically established intramammary infections, especially those caused by biofilm‐forming bacterial pathogens. Under such circumstances, it may be unrealistic to expect nonantibiotic agents to fully replace antibiotics [88, 89]. Hence, it will be more pragmatic to consider therapeutic nonantibiotic alternatives as adjuncts that augment treatment efficacy or shorten antibiotic courses, rather than direct substitutes for antibiotics in severe clinical mastitis.

**FIGURE 7:**
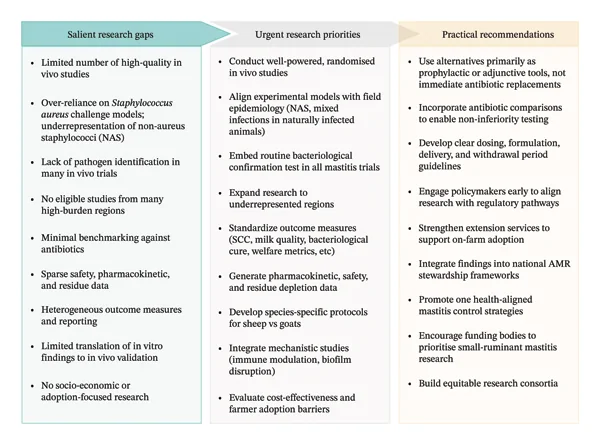
Summary of key insights from the current review based on research gaps, priorities, and practical recommendations.

To consolidate the implications of our findings and guide future work, Figure 7 summarizes the major research gaps, urgent priorities, and practical recommendations emerging from this scoping review.

## 5. Study Limitations

Despite many useful insights, several inherent limitations must be acknowledged. A general limitation of scoping reviews is the analytical depth [90], especially with a small number of studies with frequent heterogeneity in methodological approaches, outcome measures, and data reporting, as observed in this study. The limited number of comparative in vivo studies and the high degree of methodological heterogeneity preclude a definite meta‐analysis. Consequently, absolute conclusions regarding effectiveness of the alternatives could not be drawn until standardized protocols are applied across a broader range of high‐powered studies. Additionally, we could not translate non‐English publications, and the search strategy was not peer‐reviewed by a librarian or information specialist. Nonexperimental studies, including traditional and ethnoveterinary results, which may include important information, were not included. Therefore, it is possible that some relevant studies were excluded or inadvertently missed in this study. Nevertheless, we believe that our review provides comprehensive coverage of available evidence, and the charted data contain key details that validate all included studies, which were used to provide an early map of candidate alternatives and experimental approaches.

## 6. Conclusions

In summary, this scoping review provides the most recent and comprehensive synthesis of nonantibiotic alternatives for mastitis control in small ruminants. While the present evidence base is limited, the findings offer cautious optimism with the phytochemical, probiotic, and immunostimulatory candidates identified. Although many more alternatives can be explored, but to translate promise into practice, there is a need to move from mere exploratory to more standardized and context‐sensitive trials that reflect spatial, epidemiological, biological, socioeconomic, and cultural realities of small‐ruminant farming, accompanied by safety and residue evaluations. Future studies will also benefit from incorporating standard antibiotic comparators to better contextualize the efficacy of new alternatives. Achieving these goals will require deliberate action plans collectively developed by research councils, scientists, regulatory bodies, and small‐ruminant farmers. Since high‐level research rarely develops in isolation, policymakers and funding agencies committed to antimicrobial stewardship and livestock health will also have to support this translational agenda so that, in the near future, small‐ruminant producers can access validated, safe, and affordable tools that reduce reliance on antibiotics while protecting animal health and public health. With such strategic investment and interdisciplinary collaboration, we believe that, over time, nonantibiotic alternatives could become valuable tools in the global effort to promote sustainable, responsible mastitis management.

## Author Contributions

Mahmoud Eltholth and Ismail Ayoade Odetokun: conceptualized the study and acquired funding. Ismail Ayoade Odetokun, Mahmoud Eltholth, Afisu Basiru, and Damilare O. Famakinde: designed the methodology. Damilare O. Famakinde: acquired, validated, analyzed, interpreted, and visualized the data and wrote the original draft of the manuscript. Rodhiat Oyinlola Ade‐Yusuf, Mutiat Adenike Adetona, and Afisu Basiru: searched and screened study records for eligibility. Damilare O. Famakinde, Jennifer Cole, Ismail Ayoade Odetokun, Mabrouk Elsabagh, Elena Garcia‐Fruitós, Anna Arís, and Mahmoud Eltholth: revised and edited the manuscript for intellectual enhancement. Damilare O. Famakinde had full access to all the data in this study and takes complete responsibility for the integrity of the data and the accuracy of the data analysis.

## Funding

This work was part of the BAC4RumA project (ID: 110337‐002) funded by the International Development Research Centre (IDRC), Canada, and the Global AMR Innovation Fund (GAMRIF), part of the UK Government’s Department of Health and Social Care (DHSC). The funders were not involved in report writing, data collection, analysis, and interpretation, or the decision to submit the report for publication.

## Disclosure

The lead author affirms that this manuscript is an honest, accurate, and transparent account of the study being reported; that no important aspects of the study have been omitted; and that any discrepancies from the study as planned (and, if relevant, registered) have been explained. All authors have read and approved the final version of the manuscript.

## Conflicts of Interest

The authors declare no conflicts of interest.

## Supporting Information

Additional supporting information can be found online in the Supporting Information section.

## Supporting information


**Supporting Information 1** Search terms used for each database and search engine.

**Supporting Information 2** Metadata containing extracted data from each included study and quality assessment scores.

## Data Availability

All data generated are included in this article and its supporting information. All R codes used for data visualization can be accessed via GitHub at https://github.com/DamiF25/BAC4RumA_SmaRT.git.
