# Supplementary material for: Nonantibiotic Alternatives for Mastitis in Small Ruminants: A Scoping Review of Evidence, Gaps, and Translational Challenges
Source: Vet Med Int. 2026 Sep 21;2026:6174170. doi: 10.1155/vmi/6174170 (PMC13594565; doi:10.1155/vmi/6174170)
Supplement: Supplementary file 1 [file VMI-2026-6174170-s001.pdf]

SEARCH TERMS USED FOR EACH DATABASE AND SEARCH ENGINE

PubMed

Search Query:

Keywords and Boolean operators were employed to target studies on alternatives to antibiotics, mastitis, and small ruminants.

"natural" OR "feed additives" OR "probiotic" OR "prebiotic" OR "bacteriophage" OR "plants" "extract" OR "Herbs" OR "immunomodulator" OR "nano" OR "Aminopeptides"

AND

"Mastitis" OR "udder" OR "mammary gland " OR "mammary infection" OR "ovine mastitis" OR "caprine mastitis"

AND

"Sheep" OR "Goats"

Expected Output: High-quality results on alternatives to antibiotics for mastitis in small ruminants

Scopus

Search Query:

Modified Boolean operators were adapted to Scopus-specific requirements.

Example:

TITLE-ABS-KEY (natural* OR additive* OR probiotic* OR prebiotic OR bacteriophage OR plant* OR extract* OR herbs OR immunomodulator OR nano OR aminopeptide) AND TITLE-ABS-KEY (mastitis* OR udder OR "mammary gland" OR "mammary infection" OR "ovine mastitis" OR "caprine mastitis") AND TITLE-ABS-KEY (sheep OR goat) AND PUBYEAR > 2014 AND PUBYEAR < 2026

Google Scholar

Search Strategy:

Key terms were combined to yield broader search results and grey literature.

Example: **natural extract, mastitis, sheep, goat, herbs, probiotic, prebiotic, udder**

Web of Science

Search query:

Modified Boolean operators were adapted to the Web of Science requirements.

("natural" OR "feed additives" OR "probiotic" OR "prebiotic" OR "bacteriophage" OR "plant extract*" OR "herb*" OR "immunomodulator*" OR "nano*" OR "aminopeptide*")

AND ("mastitis" OR "udder" OR "mammary gland" OR "mammary infection" OR "ovine mastitis" OR "caprine mastitis")

AND ("sheep" OR "goat*")

Embase

Search query:

Modified Boolean operators were adapted to the Embase requirement.

('natural' OR 'feed additives' OR 'probiotic' OR 'prebiotic' OR 'bacteriophage' OR 'plant extract' OR 'herbs' OR 'immunomodulator' OR 'nano' OR 'aminopeptides')

AND

('mastitis' OR 'udder' OR 'mammary gland' OR 'mammary infection' OR 'ovine mastitis' OR 'caprine mastitis')

AND

('sheep' OR 'goats')
